# Thoughts, beliefs and concepts concerning infectious childhood diseases of physicians practicing homeopathic, anthroposophic and conventional medicine – a qualitative study

**DOI:** 10.1186/s12906-021-03216-2

**Published:** 2021-01-26

**Authors:** N. Mittring-Junghans, C. Holmberg, C. M. Witt, M. Teut

**Affiliations:** 1 Institute for Social Medicine, Epidemiology and Health Economics, Charité – Universitätsmedizin, Luisenstr. 57, 10117 Berlin, Germany; 2 Institute of Social Medicine and Epidemiology, Medical School Theodor Fontane, Brandenburg/Havel, Germany

**Keywords:** measles, rubella, mumps, chickenpox, vaccination, homeopathy, anthroposophy, qualitative research

## Abstract

**Background:**

Physicians who include complementary medicine in their practice are thought to have an understanding of health and disease different from that of colleagues practicing conventional medicine. The aim of this study was to identify and compare the thoughts and concepts concerning infectious childhood diseases (measles, mumps, rubella, chickenpox, pertussis and scarlet fever) of physicians practicing homeopathic, anthroposophic and conventional medicine.

**Methods:**

This qualitative study used semistructured interviews. Participating physicians were either general practitioners or pediatricians. Data collection and analysis were guided by a grounded theory approach.

**Results:**

Eighteen physicians were interviewed (6 homeopathic, 6 anthroposophic and 6 conventional). All physicians agreed that while many classic infectious childhood diseases such as measles, mumps and rubella are rarely observed today, other diseases, such as chickenpox and scarlet fever, are still commonly diagnosed. All interviewed physicians vaccinated against childhood diseases. A core concern for physicians practicing conventional medicine was the risk of complications of the diseases. Therefore, it was considered essential for them to advise their patients to strictly follow the vaccination schedule. Homeopathic-oriented physicians viewed acute disease as a biological process necessary to strengthen health, fortify the immune system and increase resistance to chronic disease. They tended to treat infectious childhood diseases with homeopathic remedies and administered available vaccines as part of individual decision-making approaches with parents. For anthroposophic-oriented physicians, infectious childhood diseases were considered a crucial factor in the psychosocial growth of children. They tended to treat these diseases with anthroposophic medicine and underlined the importance of the family’s resources. Informing parents about the potential benefits and risks of vaccination was considered important.

All physicians agreed that parent-delivered loving care of a sick child could benefit the parent-child relationship. Additionally, all recognized that existing working conditions hindered parents from providing such care for longer durations of time.

**Conclusions:**

The interviewed physicians agreed that vaccines are an important aspect of modern pediatrics. They differed in their approach regarding when and what to vaccinate against. The different conceptual understandings of infectious childhood diseases influenced this decision-making. A survey with a larger sample would be needed to verify these observations.

## Background

According to the WHO SAGE working group, vaccine hesitancy refers to the delay in acceptance or refusal of vaccines despite the availability of vaccination services. Vaccine hesitancy is complex and context-specific, varying across time, place and vaccines and is influenced by factors such as complacency, convenience and confidence. Vaccine hesitancy can be understood as the behavior that results from the decision-making process and reflects numerous factors that may influence the decision to accept some or all vaccines according to the recommended schedule [1].

In Western countries, a conceptual discussion exists between complementary and alternative medicine practitioners and physicians and conventional physicians on the use of vaccinations to prevent classical childhood diseases such as mumps, measles, rubella, chickenpox and pertussis.

In addition to misinformed and skeptical parents [2–4], the influence of physicians with a specialization in anthroposophic or homeopathic medicine on parents has been described as one reason for low vaccination rates [5–7]. In Germany, approximately 7,000 physicians have a specialization in homeopathic medicine [8] and approximately 6,000 physicians are predominantly practicing anthroposophic medicine [9].

In 1996, a survey in Germany among general practitioners practicing homeopathy showed that homeopathic doctors are not per se against vaccination but are more critical in terms of when to vaccinate and what to vaccinate against [10]. A subsequent representative population-based cross-sectional study in 2006 among German pediatricians with and without homeopathic specialization confirmed these results [11]. European studies have shown that the vaccination coverage for measles, mumps and rubella in anthroposophic Steiner schools are lower than that in reference schools [12–14]. A focus group study from the Netherlands showed that anthroposophic childhood vaccine providers spent more time communicating with parents concerning the benefits and risks of vaccinations and often adapt the immunization scheme to the individual case [15].

Due to the differing underlying philosophical approaches to disease among biomedicine, anthroposophic, and homeopathic medicine, physicians also have different thoughts and beliefs about the underlying concepts of infectious childhood disease, which may influence their views on vaccination and particular vaccination behaviors.

Therefore, the objective of this study was to investigate the concepts, thoughts and beliefs of general practitioners and pediatricians in Germany practicing conventional, homeopathic or anthroposophic medicine concerning the classic childhood illnesses measles, mumps, rubella, chickenpox, pertussis and scarlet fever.

## Methods

### Design

A qualitative interview study was conducted [16]. Data collection and analyses were based on the methodological concept of grounded theory [17, 18] to generate hypotheses based on the data [19]. The study was approved by the ethics committee of the Charité –Universitätsmedizin Berlin (EA1/078/09, 25/05/2009).

### Sample

We assumed that physicians who were representatives of their professional associations (e.g., conventional, homeopathic, and anthroposophic medicine) were the most appropriate for the interview to capture typical opinions in the respective fields. The physicians had to meet the following inclusion criteria:

- Physicians practicing conventional medicine: female or male pediatric practitioners without further training in complementary medicine
- Physicians practicing homeopathic medicine: female or male pediatric or general practitioners with a homeopathy diploma of the “DZVhÄ” (Deutscher Zentralverein homöopathischer Ärzte)
- Physicians practicing anthroposophic medicine: female or male pediatric or general practitioners with an additional qualification in anthroposophic medicine of the “GAÄD” (Gesellschaft Anthroposophischer Ärzte in Deutschland)

### Informed consent

- Adequate knowledge of the German language

Thus, the selection was performed using the respective professional organizations. Participating physicians were either recommended by professional organizations for pediatricians, homeopathy or anthroposophic medicine (n=8) or by participants (snowball sampling, n=10).

In the context of the grounded theory approach, a theoretical saturation of data was sought and recruitment was conducted accordingly [18]. Thus, interview partners were sought until theoretical saturation was achieved among the three physician groups. The potential interview participants were contacted via email or telephone. If they agreed to participate, an interview appointment was scheduled.

### Interview guideline

Based on literature analyses of established textbooks of conventional pediatrics,[20–22] homeopathic pediatrics [23–25] and anthroposophic pediatrics [26, 27], hermeneutic content analysis was conducted to filter out the most important aspects and statements concerning the study objective. The results were used to develop questions for a semistructured interview guideline (see Table 1).

**Table 1:** First interview guide

Interview guide	
**Initial questions:** How do you deal with infectious childhood diseases in your daily practice? or Please describe your personal understanding of infectious childhood diseases. or In your opinion, which diseases belong to the infectious childhood diseases?	
**Complements** (themes that should be requested if not already approached by the interviewee)**:** • Infantile immune system • Chronological correlation • Meaning of fever • Influence of fever on the development of a child • Risks • Association with diseases in later life • Impact on the mental and physical development of the child • Family life • Prevention	

### Data collection

The interviews were conducted face-to-face in each physician’s practice and were led by a trained qualitative researcher (NM). All the participants provided informed consent. The interviews were digitally recorded, and the researcher wrote a short summary after each interview.

### Data analyses

The interviews were transcribed verbatim. Each interview was pseudonymized. Analyses followed a grounded theory approach assisted by Atlas/ti® software [28]. After the first interviews were transcribed and coded, the subsequent interviews were conducted such that questions developed from the first round of results were included to gather new findings from the interviews. Data collection and theory generation were alternated; the analysis process occurred in a triadic and circular constant comparative manner [18, 29]. Regarding the theoretical framework of the grounded theory approach, theoretic saturation was reached with six interviews per group [19].

Written memos during the coding and analysis process supported the analyses and results. The analyses and results were regularly discussed in the research team and in a qualitative research group to ensure intersubjectivity and grounding of results in the material.

## Results

### Sample

Eighteen physicians were interviewed from all over Germany, including eleven male and seven female physicians (for physicians’ characteristics, see Table 2). The interviews lasted from 14:16 to 63:02 min (on average, 40:09 min ± 24:23 min). Twelve physicians worked in practices participating in the statutory health insurance system, and six physicians had private practices.

**Table 2:** Characteristics of the interview partners

	Conventional physicians	Homeopathic physicians	Anthroposophic physicians
**Sex (male:female)**	4:2	4:2	3:3
**Age (mean ± SD)**	51.2 ± 6.2	53.2 ± 9.8	50.3 ± 6.2
**Qualification:**			
**• General practitioner**	-	4	5
**• Pediatrician**	6	1	1
**• Internal medicine specialist**	-	1	-
**Reimbursement system:**			
**• Statutory health system**	6	5	5
**• Private practice**	-	1	1

### Results of the physicians practicing conventional medicine

We interviewed six conventional pediatricians who all worked in practices participating in the German statutory health insurance system (see Table 2). In these practices, classic infectious childhood diseases were observed very rarely because of the quite high vaccination rates in Germany, a situation that was considered to be very positive by those physicians.

#### General understanding of illness

For the conventional pediatricians, diseases, and particularly infections in childhood, played an important role in the build-up and maturation of the immune system. However, due to potential complications, infectious childhood diseases were also viewed as risky, stressful, and potentially harmful to the child’s development.

#### Role of fever

Fever was, for the most part, characterized as a positive and physiological immunological response and played an important role in the development of the body’s defense mechanisms because it trained the immune system. Most pediatricians recommended to parents that fevers of 39 °C (102.2 °F) or above should be lowered. Indeed, they observed that parents felt generally so stressed and worried about a febrile child that they often made efforts to lower the fever even if it was lower than 39 °C.

#### Burden on the family

Overall, the need for emotional security among parents represented an important theme in the interviews with the conventional pediatricians. It was an important part of the physicians’ daily routine to calm parents who felt threatened and burdened by a sick child. The physicians emphasized that, generally, attention and closeness of the parents played an essential role in a sick child, but they also observed that parents currently have little time to care for their children because they had to work and because they were not prepared to assume the responsibility.

#### Infectious childhood disease as a risk

Altogether, the risks and complications of infectious childhood diseases were given priority in the interviews. Experiencing these diseases entailed many risks due to potentially complicated consequences (e.g., the development of measles encephalitis). To develop a robust and healthy immune system, low-risk infections were deemed sufficient and posed less risk.

#### Vaccination as a positive prevention measure

All of the conventional physicians supported vaccination against [most - some were against Varicella] infectious childhood diseases and were confident of its necessity.

*“I say such vaccination in the end is a sort of life insurance! I insure [patients] against something that I hope will not occur. You have life insurance for yourself, and perhaps even for your child, and the vaccination is insurance, too.”—P 14, conventional physician.*

They reported having very good experiences with the officially recommended German vaccination program (STIKO-Empfehlungen) [29] and considered that these vaccinations protected the child and family. Furthermore, the preventive role of vaccinations on a population level for herd immunity was stressed. Additionally, they considered the prevention of childhood diseases as essential for social and economic reasons because the vaccinations prevented the parents from long periods of absence from work. Some interview participants in this group did not agree with the administration of the varicella vaccination because, in their opinion, this illness progressed without complications among healthy children and, therefore, vaccination would not be needed. They also critically discussed the impact of the economic interests mentioned above for the implementation of this vaccination.

### Results of the physicians practicing homeopathic medicine

Six physicians (4 pediatricians, 1 internal medicine specialist, and 1 general practitioner) with a specialization in homeopathy were interviewed (Table 2). Except for one, all of them worked in private practices.

#### Meaning of infectious childhood disease

Generally, the homeopathic physicians believed that experiencing diseases, including infectious childhood diseases, was an important and elemental part of life. Five of the six participants also discussed a biological dimension in the experience of infectious childhood diseases.

*“A human being can always get sick. Childhood diseases are only a part of it.”—P 03, homeopathic physician*

The physicians concurred that, during those diseases, important mental and physical maturation processes occur in the child. These diseases were considered important for the development of the child’s immune system, and it was discussed that experiencing a full course of these diseases could act as a protective factor against developing a potentially wide range of chronic diseases, including future psychiatric diseases.

In this context, the physicians often mentioned the importance of the homeopathic concept of the development of disease-typical eczemas. The suppression of those eczemas (e.g., by corticosteroids) might facilitate the development of chronic conditions according to a homeopathic theory termed Hering’s law [30]. Generally, the physicians reported seeing mild courses of childhood diseases with only a few complications between the second year of age and puberty.

#### Influence on the parent-child relationship

The physicians considered the impact of childhood diseases on the parent-child relationship to be very important because the joint experience of coping with such a disease strengthened the relationship and trust between them. The interviewed physicians believed that parents have a responsibility to support and care for their sick child, as well as to provide time and a calm and loving environment. The physicians perceived themselves as advisers and attendants to the family and aimed to train the parents to treat a sick child competently. They offered the critique that currently parents do not spend sufficient time with their children because of their jobs and other reasons.

*“Well, I think it plays a certain role in how secure a child feels in the family, (...) how tense the parents are. Unfortunately, the parents in the practice often say: we both have to work (...), and the child – it is not possible, I can´t stay at home for two weeks, (...) s/he has to recover quickly. (...) that is simply the problem in modern times.”—P 01, homeopathic physician.*

#### Importance of fever

From the homeopathic physicians’ point of view, fever was understood as an important physical symptom and expression of a healthy immune defense and self-healing-effort, which should only be lowered very cautiously. Antipyretic treatment was regarded with skepticism because fever was also considered an important clinical symptom that could support understanding of the course of disease and support finding the right homeopathic remedy.

#### Individual decision on vaccination

Predominantly, the vaccination of infectious childhood diseases was discussed with great seriousness. It was important for the physicians to consider about vaccinations in a differentiated and individualized manner.

*“Vaccinations (...) are no insurance against disease. You should truly think carefully about what to vaccinate against and when.”—P 01, homeopathic physician.*

Except for one, all the physicians vaccinated their patients. Additionally, they thoroughly informed the parents about the advantages and disadvantages and aimed to support an individual and autonomous parental decision. Generally, they tended to vaccinate children later than the timeline suggested by German official recommendations (e.g., when a child had learned to walk because the brain´s development had reached an important threshold) and preferred single over combined vaccine products. Overall, the homeopathic physicians called for more long-term studies about the side effects, efficacy, and tolerance of vaccinations. It became apparent that one physician who was specialized in homeopathy and worked in a practice belonging to the statutory health insurance system had different opinions. Discussing infectious childhood diseases, he saw fewer advantages and higher risks of complications when children were not vaccinated as recommended. For him, vaccinations played a more relevant and useful role than that for the other homeopathic physicians.

### Results of the physicians practicing anthroposophic medicine

Six physicians with a specialization in anthroposophic medicine were interviewed. Except for one physician, all of the participants worked in practices participating in the statutory health system. They had little contact with infectious childhood diseases except for chickenpox and scarlet fever.

#### Infectious childhood disease as a potential chance for development

For the anthroposophic physicians, classic childhood diseases were important chances to facilitate the maturation of the child´s individuality and immune system. In their opinions, the experience of these diseases could help to better connect the physical and mental parts of the body. Furthermore, diseases were generally considered to be important periods of rest, allowing the body to return to a state of physical and spiritual balance.

#### Role of the family

The support of the family’s competence to care for a sick child was a main point of argument. Anthroposophic physicians viewed themselves as family educators and mentors. They all organized information and education events where parents could learn about self-help strategies and natural home remedies (e.g., how to apply compresses to lower fever). For the physicians supporting the psychological resources of the parents, factors such as stable surroundings and structures, a calm atmosphere, perseverance, and trust were reported to be crucial.

#### Fever as an activity of the personality

Fever was considered to be a positive symptom and sign of activity of the child’s personality. Fever was thought to play a very important role in the individualization and development of the child. It was considered to be essential to permit this physical self-regulation and to only suppress fever in rare cases, depending on the general condition of the child. The physicians observed that parents currently have much fear of fever and that antipyretic treatment would often serve as reassurance for them. From the anthroposophic physicians’ point of view, fever was essential for health maintenance and might protect against the development of chronic diseases, such as allergies. Therefore, the suppression of a symptom of disease was discussed as a lost chance for development.

#### Embedding of the vaccination decision into the family’s situation

In general, the physicians practicing anthroposophic medicine tended to vaccinate later than the official German STIKO recommendations [29], and their vaccination practice was more individualized and differentiated. Anthroposophic physicians believed that their vaccination practice was better customized to meet the parents´ needs. It was essential for them to advise the parents about vaccinations in a detailed manner. They also offered information meetings about fever and vaccinations and expected a proper and reflective decision from the parents. The individual resources and possibilities of a family played an important role in this decision (e.g., if the parents had sufficient time to care for a sick child).

*“I think it is very important that they [the parents] know what they’re getting involved with. In addition, I don´t think that you can provide these diseases. However, rather the parents have to face up to them actively.”—P 12, anthroposophic physician.*

## Discussion

This qualitative study investigated the concepts and beliefs toward infectious childhood diseases among physicians practicing conventional, homeopathy and anthroposophic medicine.

While the conventional physicians predominantly saw risks in the natural course of these diseases and highlighted the advantages of vaccination, the physicians with a specialization in homeopathy and anthroposophic medicine assumed that these diseases could have positive influences on the child’s, as well as the family’s, overall development.

### Limitations

Only a small number of physicians were interviewed for our qualitative study, and the interview data served for generating our hypotheses of this research report. A quantitative survey with a larger sample would be needed to verify our observations as next step.

Some of the interview questions that were derived from the literature (e.g., the necessity of childhood diseases for the maturation of a child) were conceived to be skeptical, especially by the conventional physicians, possibly influencing the interview situation and reporting. Another aspect to discuss is the composition of the pool of interview participants. According to our research question and the used method of grounded theory, it was important for our study to select typical representatives of the different therapeutic directions who mainly treated children. In all groups, we primarily selected pediatricians; however, in the groups of the physicians specialized in homeopathic and anthroposophic medicine, we also interviewed general practitioners and a specialist in internal medicine. This is consistent with the reality of the healthcare system in Germany, where those additionally trained physicians also treat many children. The research question exploring the thoughts and beliefs of physicians with different specializations required a qualitative study design to allow for in-depth insight into the underlying theories [16].

In the interviews, we found relevant differences in the attitudes between the groups but also some similarities.

### Similarities

For all the physicians, the major meaning of infantile infectious diseases was the maturation of the immune system. Fever was seen as an important and useful reaction of the immune system to fight against disease and was generally welcomed, a finding that is also described in the literature [20, 26, 31, 32]. Furthermore, all the physicians reported that they observed insecurity and substantially increased fears in parents concerning a sick child with fever. Presently, many parents are not fully prepared to care for a sick child (e.g., due to a lack of time, patience or because they have to work). Therefore, the interview participants all spent significant time calming and educating parents. Several studies have reported the intense fear of fever and uncertainty shared by many parents [33] and investigated influencing factors (e.g., socioeconomic status or ethnic background) [34–36]. Therefore, a very important responsibility in the daily work of physicians is to meet the parents´ needs for adequate information and education about illness, fever, and the adequate use of antipyretic treatments [31, 37, 38].

A very important similarity in our interviews indicated that all the physicians generally offered vaccinations against childhood diseases to parents, confirming the results from the mentioned studies about vaccination practices among conventional and homeopathic physicians [10, 11].

### Differences

Regarding the differences among the three groups, the main difference between the physician groups concerned the understanding and prevention of diseases. For the conventional physicians, the positive influence of disease on the immune system and the child’s development was limited to low-risk infectious diseases. They believed that classic childhood illnesses were associated with too many risks and complications and must be prevented, as also recommended widely in the German pediatric literature [20, 21, 39]. By contrast, the physicians specializing in homeopathy (except for one pediatrician described above) and anthroposophic medicine saw also positive aspects in going through a full course of a classic childhood disease. The classic childhood diseases were also discussed to play a special role in the maturation of the immune system and the child’s personality. In this respect, for the anthroposophic physicians, the resources and social circumstances of an individual child and his or her family were crucial aspects in deciding whether vaccination against such diseases was associated with positive aspects. For the physicians specialized in homeopathy, the acceptance of the natural course of diseases had priority. Both physician groups emphasized the importance of informing parents about vaccinations and classic childhood diseases. Studies have shown that parents have insecurities regarding vaccinations and want to be informed broadly by their pediatrician [40, 41].

In the interviews with the conventional pediatricians, fever played an important role in the physical fight against acute disease; however, physicians practicing anthroposophic medicine or homeopathy believed that fever could provide a long-term protective factor against chronic diseases. Studies describing a possible association of the frequent application of antipyretics in childhood with an increased risk for allergies and asthma [14, 42] stand in contrast to other studies, which have not confirmed this theory [43, 44].

Furthermore, the association of fever as an activity of the child’s personality and a possible protective factor against allergies can be found in several studies concerning anthroposophic medicine [45].

All the interviewed physicians recommended vaccination against classic childhood diseases to parents but in different ways. The conventional pediatricians mainly followed the official German vaccination recommendations [30]. However, as already investigated in other studies, the interview participants specializing in anthroposophic medicine or homeopathy modified this recommendation scheme and often vaccinated children later and in a more individualized way according to the parents´ wishes [10, 11, 15].

Altogether, the conventional physicians saw many risks in the natural course of classic childhood illnesses and appreciated vaccinations as providing relief for the child and family. By contrast, the physicians trained in homeopathy or anthroposophic medicine expected more prominent unknown risks because of vaccinations, due to suppression of the natural course of disease. Different concepts of disease lead to differences in the perceptions of risk and the benefit of prevention measures. While prevention in medicine aims to eliminate classic childhood diseases, anthroposophic and homeopathic literature also describes positive aspects of undergoing these diseases for childhood development [24, 26, 46]. Table 3 shows the summary and a comparison of the most important results.

**Table 3:** Similarities and differences of concepts towards classical childhood diseases - results from the interviewed physicians

	Physicians practicing …		
	Conventional medicine	Homeopathic medicine	Anthroposophic medicine
**Similarities:**			
*Meaning of infectious diseases*	Maturation of immune system		
*Fever*	Important and useful reaction of the immune system Parents: insecurity and fears concerning fever		
*Vaccination*	All physicians offered vaccinations to the families		
**Differences:**			
*Importance of infectious childhood diseases and perception of risk*	Too many risks/ complications, trivial infectious disease are sufficient	Acceptance of the natural course of all diseases	Important role in the maturation of the child’s development and maturation of the immune system
*Consultation concerning vaccinations*	Followed the official German vaccination recommendations (except varicella)	Modified recommendation scheme, later and individualized, adjusted to the parents´ wishes	Modified recommendation scheme, later and individualized, adjusted to the parents´ wishes
*Fever*	Physical fight against disease	Fever as a possible long-term protective factor against chronic diseases	Fever as an activity of the child’s personality and a possible long-term protective factor against chronic diseases

WHO declared vaccine hesitancy as one of the ten threats to global health [47]. The results of our research indicate that, at least in industrialized countries, there might be conceptual and sociocultural differences in views of health and disease when comparing physicians practicing homeopathy or anthroposophic medicine with those practicing conventional medicine only. To address the current problem of vaccine hesitancy more effectively, discussions should also address the problem of different concepts of health and disease on an individual but also a public health level. Within the COVID-19 pandemic it will also be interesting to observe the development of the acceptance of infectious childhood vaccines, as getting through this pandemic might change the acceptance in the population and the attitudes of physicians towards the mentioned vaccines.

## Conclusion

All the interviewed physicians vaccinated their patients against childhood diseases. The anthroposophic and homeopathic physicians in our sample conceptually perceived the experience of the natural processes of disease of infectious childhood diseases as important factors for the biological and mental maturation of the children. They saw a purpose in the experience of a full immune response to infectious childhood diseases. Physicians with a conventional-only background predominantly saw the risks in the natural course of these diseases and stressed the advantages of preventing the diseases by vaccination.

Different core concepts toward the conceptual infectious childhood diseases combined with the different backgrounds of treated families seem to influence how and which preventive and therapeutic interventions are administered. Furthermore, the individual situations of families and their concepts of health and disease within the society play a very important role in the discussion about childhood disease and vaccination. The thoughts, beliefs and behavior of physicians toward vaccination and treatment of fever in children cannot be understood and changed without the knowledge of underlying concepts and the roles of social backgrounds.

## Data Availability

The datasets used and/or analysed during the current study are available from the corresponding author on reasonable request.

## Abbreviations

WHO: World Health Organization
WHO Sage: World Health Organization Study on global AGEing and adult health
SD: Standard Deviation

## References

[1] Report of the SAGE working group on vaccine hesitancy. https://www.who.int/immunization/sage/meetings/2014/october/SAGE_working_group_revised_report_vaccine_hesitancy.pdf?ua=1

[2] Siedler A, Mankertz A, Feil F, Ahlmeyer G, Hornig A, Kirchner M (2011). Closer to the goal: efforts in measles elimination in Germany 2010. J Infect Dis.

[3] Wichmann O, Siedler A, Sagebiel D, Hellenbrand W, Santibanez S, Mankertz A, et al. Further efforts needed to achieve measles elimination in Germany: results of an outbreak investigation. Bull World Health Organ. 2009:87. 10.2471/BLT.07.050187 PMC2636188 19274362

[4] Schönberger K, Grote V, von Kreis R, Kaltes H (2009). Risk factors for delayed or missed measles vaccination in young children. Bundesgesundheitsblatt Gesundheitsforschung Gesundheitsschutz.

[5] Jungbauer-Gans M, Kriwy P (2003). Influence exercised by physicians on the vaccination rate. Gesundheitswesen.

[6] Meyer C, Reiter S (2004). Vaccine opponents and sceptics. History, background, arguments, interaction. Bundesgesundheitsbl Gesundheitsforsch Gesundheitsschutz.

[7] Ernst E (2001). Rise in popularity of complementary and alternative medicine: reasons and consequences for vaccination. Vaccine.

[8] Linde K, Buitkamp M, Schneider A, Moos S, Böcken J, Braun B, Repschläger U (2012). Naturheilverfahren, komplementäre und alternative Therapien. Gesundheitsmonitor 2012.

[9] Marstedt G, Moebus S, Bundes G d (2002). Inanspruchnahme alternativer Methoden in der Medizin. Robert-Koch-Institut.

[10] Lehrke P, Nuebling M, Hofmann F (2001). Attitudes of homoeopathic physicians towards vaccination. Vaccine.

[11] Schmidt J, Bruns R (2010). Pediatricians in Private Practice and their Attitude towards Vaccination: A Comparison between Homeopaths and Non-Homeopaths in Germany. Eur J Int Med.

[12] Alm JS, Swartz J, Lilia G, Scheynius A, Pershagen G (1999). Atopy in children of families with an anthroposophic lifestyle. Lancet.

[13] Klomp JH, van Lier A, Ruijs WL (2015). Vaccination coverage for measles, mumps and rubella in anthroposophical schools in Gelderland, The Netherlands. Eur J Public Health.

[14] Floistrup H, Schwartz J, Bergström A, Alm JS, Scheynius A, van Hage M (2006). Allergic disease and sensitization in Steiner school children. J Allergy Clin Immunol.

[15] Mollema L, Staal JM, van Steinbergen JE, Pulsen TGWM, de Melker HE (2012). An exploratory qualitative assessment of factors influencing childhood vaccine providers' intention to recommend immunization in the Netherlands. BMC Public Health.

[16] Green J, Thorogood N (2004). Qualitative Methods for Health Research.

[17] Glaser BG, Strauss AL (1998). Grounded theory. Strategien qualitativer Forschung. Hans Huber Programmbereich Pflege.

[18] Glaser BG, Strauss AL (1999). The Discovery of Grounded Theory: Strategies for Qualitative Research.

[19] Lamnek S (2005). Qualitative Sozialforschung.

[20] Bialek R, Sitzmann FC, Bartmann P (2007). Infektionskrankheiten. Pädiatrie.

[21] Muntau A (2007). Intensivkurs Pädiatrie.

[22] Koletzko B, Harnack GA, Belohradsky BH (2005). Kinderheilkunde und Jugendmedizin.

[23] Hirte M, Lucae C, Pfeiffer H, Bonath T (2007). Infektionskrankheiten. Homöopathie in der Kinder- und Jugendmedizin.

[24] Graf FP (2003). Homöopathie und die Gesunderhaltung von Kindern und Jugendlichen.

[25] Voegeli A (1993). Homöopathische Therapie der Kinderkrankheiten.

[26] Soldner G, Stellmann HM (2007). Individuelle Pädiatrie. Leibliche, seelische und geistige Aspekte in Diagnostik und Beratung. Anthroposophisch-homöopathische Therapie. 3rd ed.

[27] Tautz C (2000). Kinderkrankheiten - Krankheiten im Kindesalter? Schulmedizinische und anthroposophisch erweiterte Perspektiven. Stuttgart.

[28] Muhr T, Boehm A, Mengel A, Muhr T (1994). ATLAS/ti: ein Werkzeug für die Textinterpretation. Texte verstehen: Konzepte, Methoden, Werkzeuge.

[29] Broom A (2005). Using qualitative interviews in CAM research: a guide to study design, data collection and data analysis. Complement Ther Med.

[30] Robert-Koch-Institut. Empfehlungen der Ständigen Impfkommission (STIKO) am Robert Koch-Institut/August 2013. Epidemiologisches Bulletin. 2013:34.

[31] Sullivan JE, Farrar HC (2011). Fever and antipyretic use in children. Pediatrics.

[32] Sarrell M, Cohen HA, Kahan E (2002). Physicians', nurses', and parents' attitudes to and knowledge about fever in early childhood. Patient Educ Couns.

[33] Schmitt BD (1980). Fever phobia: misconceptions of parents about fevers. Am J Dis Child.

[34] Leiser D, Doitsch E, Meyer J (1996). Mothers' lay models of the causes and treatment of fever. Soc Sci Med.

[35] Taveras EM, Durousseau S, Flores G (2004). Parents' beliefs and practices regarding childhood fever: a study of a multiethnic and socioeconomically diverse sample of parents. Pediatr Emerg Care.

[36] Tessler H, Gorodischer R, Press J, Bilenko N (2008). Unrealistic concerns about fever in children: the influence of cultural-ethnic and sociodemographic factors. Isr Med Assoc J.

[37] Kai J (1996). Parents' difficulties and information needs in coping with acute illness in preschool children: a qualitative study. BMJ.

[38] Langer T, Pfeifer M, Soenmez A, Kalitzkus V, Wilm S, Schnepp W (2013). Activation of the maternal care giving system by childhood fever -- a qualitative study of the experiences made by mothers with a German or a Turkish background in the care of their children. BMC Fam Pract.

[39] Sitzmann FC, Bartmann P (2007). Pädiatrie Duale Reihe.

[40] Harmsen IA, Ruiter RAC, Paulussen TGW, Mollema L, Kok G, de Melker HE (2012). Factors that influence vaccination decision-making by parents who visit an anthroposophical child welfare center: a focus group study. Adv Prev Med.

[41] Pauli J, Dilger B (2010). Wem glauben? Eine explorative Studie zu Impfentscheidungen von Kölner Eltern zwischen Internet, ärztlicher Autorität und verwandschaftlichem Vertrauen. Medizin im Kontext: Krankheit und Gesundheit in einer vernetzten Welt.

[42] Beasley R, Clayton T, Crane J, von Mutius E, Lai CKW, Montefort S (2008). Association between paracetamol use in infancy and childhood, and risk of asthma, rhinoconjunctivitis, and eczema in children aged 6-7 years: analysis from Phase Three of the ISAAC programme. Lancet.

[43] Tapiainen T, Dundee T, Möttönen M, Pokka T, Uhari M (2010). Adolescents with asthma or atopic eczema have more febrile days in early childhood: a possible explanation for the connection between paracetamol and asthma?. J Allergy Clin Immunol.

[44] Schnabel E, Heinrich J (2010). Respiratory tract infections and not paracetamol medication during infancy are associated with asthma development in childhood. J Allergy Clin Immunol.

[45] Martin DD (2016). Fever: Views in Anthroposophic Medicine and Their Scientific Validity. Evid Based Complement Alternat Med.

[46] Rosenlund H, Bergstrom A, Alm JS, Swartz J, Scheynius A, van Hage M (2009). Allergic disease and atopic sensitization in children in relation to measles vaccination and measles infection. Pediatrics.

[47] WHO: Ten threats to global health in 2019. Geneva 2019. https://www.who.int/emergencies/ten-threats-to-global-health-in-2019. Accessed 21 Feb 2020.

